# Rapid transcriptome sequencing of an invasive pest, the brown marmorated stink bug *Halyomorpha halys*

**DOI:** 10.1186/1471-2164-15-738

**Published:** 2014-08-29

**Authors:** Panagiotis Ioannidis, Yong Lu, Nikhil Kumar, Todd Creasy, Sean Daugherty, Marcus C Chibucos, Joshua Orvis, Amol Shetty, Sandra Ott, Melissa Flowers, Naomi Sengamalay, Luke J Tallon, Leslie Pick, Julie C Dunning Hotopp

**Affiliations:** Institute for Genome Sciences, University of Maryland School of Medicine, Baltimore, MD USA; Departments of Entomology and Cell Biology & Molecular Genetics, University of Maryland College Park, College Park, MD USA; Department of Microbiology and Immunology, University of Maryland School of Medicine, Baltimore, MD USA; Department of Genetic Medicine and Development, University of Geneva Medical School, Geneva, 1211 Switzerland; Gaithersburg, MD USA

**Keywords:** Brown marmorated stink bug, *Halyomorpha halys*, Transcriptome, Hemiptera, Invasive species, Lateral gene transfer, Horizontal gene transfer, Mannanase, Lysozyme

## Abstract

**Background:**

*Halyomorpha halys* (Stål) (Insecta:Hemiptera;Pentatomidae), commonly known as the Brown Marmorated Stink Bug (BMSB), is an invasive pest of the mid-Atlantic region of the United States, causing economically important damage to a wide range of crops. Native to Asia, BMSB was first observed in Allentown, PA, USA, in 1996, and this pest is now well-established throughout the US mid-Atlantic region and beyond. In addition to the serious threat BMSB poses to agriculture, BMSB has become a nuisance to homeowners, invading home gardens and congregating in large numbers in human-made structures, including homes, to overwinter. Despite its significance as an agricultural pest with limited control options, only 100 bp of BMSB sequence data was available in public databases when this project began.

**Results:**

Transcriptome sequencing was undertaken to provide a molecular resource to the research community to inform the development of pest control strategies and to provide molecular data for population genetics studies of BMSB. Using normalized, strand-specific libraries, we sequenced pools of all BMSB life stages on the Illumina HiSeq. Trinity was used to assemble 200,000 putative transcripts in >100,000 components. A novel bioinformatic method that analyzed the strand-specificity of the data reduced this to 53,071 putative transcripts from 18,573 components. By integrating multiple other data types, we narrowed this further to 13,211 representative transcripts.

**Conclusions:**

Bacterial endosymbiont genes were identified in this dataset, some of which have a copy number consistent with being lateral gene transfers between endosymbiont genomes and Hemiptera, including ankyrin-repeat related proteins, lysozyme, and mannanase. Such genes and endosymbionts may provide novel targets for BMSB-specific biocontrol. This study demonstrates the utility of strand-specific sequencing in generating shotgun transcriptomes and that rapid sequencing shotgun transcriptomes is possible without the need for extensive inbreeding to generate homozygous lines. Such sequencing can provide a rapid response to pest invasions similar to that already described for disease epidemiology.

**Electronic supplementary material:**

The online version of this article (doi:10.1186/1471-2164-15-738) contains supplementary material, which is available to authorized users.

## Background

*Halyomorpha halys* (Stål) (Insecta: Hemiptera: Pentatomidae), otherwise known as the Brown Marmorated Stink Bug (BMSB), is an invasive pest that has ravaged farms and distressed homeowners in the mid-Atlantic region of the US in recent years. It is a polyphagous insect pest that causes economically important damage to many crops including vegetables, tree fruit, field crops, and ornamentals [1].

Native to Asia, BMSB was first observed in North America in Allentown, PA, USA in 1996 [1]. BMSB is now well-established in the mid-Atlantic regions of the US and has spread to 41 different states and DC [2]. There is also evidence for established populations in Canada [3] and Switzerland [4–6]. It is a pest of tree fruit; grapes; other small fruit; row crops including vegetables, legumes and cotton; shade trees; ornamentals; and nursery crops [7–9]. BMSB crop damage reached economically significant levels during the 2010 growing season. Growers considered BMSB to be the single most important pest in the mid-Atlantic region, leading to ‘desperate measures’ including increased use of broad-spectrum pesticides to control BMSB, detracting from sustainable Integrated Pest Management practices. In keeping with this, the EPA granted emergency approval for the use of several pesticides, including one approved for use in organic farming, to prevent further economic loss in the mid-Atlantic region [10]. In addition to being an agricultural pest, many mid-Atlantic homeowners are troubled by BMSB. Unlike native stink bugs, BMSB aggregates in human-made structures, including houses, to overwinter [11]. One home owner reported removing 26,000 BMSB from his residence in the first half of 2011 [12].

BMSB has a long history of hitchhiking to new areas. The successful introduction in Allentown, PA, USA, was preceded by the successful interception of BMSB by the USDA preventing such invasions including an interception of BMSB on a 1983 flight from Japan, an interception in baggage from Korea in 1984, and eight further interceptions from China, Korea, and Japan from 1989–1998 [8]. BMSB has been unintentionally shipped from Japan to New Zealand in a used vehicle [13]. In 2005, over a dozen BMSB were recovered from a storage unit in Vallejo, Solano County, CA, that was rented by an individual who had relocated from Pennsylvania [14]. Ecological niche modelling and climate data predict that areas between 30–50 degrees latitude are at risk of invasion including Northern Europe, north-eastern North America, the northern portions of the North American west coast, southern Australia, the North Island of New Zealand as well as Angola in Africa and Uruguay in South America [8].

As summarized above, BMSB are significant agricultural pests with limited treatment options and an ability to spread, making it a particularly difficult invasive pest to manage. Over sixty researchers funded by the USDA and commodity organizations are conducting experiments aimed at better understanding the biology and ecology of this pest and to find management solutions. When this project began, only 100 bp of sequence data was available in the public database. Transcriptome sequencing was undertaken to provide a molecular resource to the research community for basic and applied research purposes. The data can be used to examine the population genetics of BMSB, including gaining a better understanding of the original invasion in Allentown, as well as subsequent invasions throughout the US and the world. Understanding these invasions will increase our knowledge, preventing future invasions. Our study demonstrates that the transcriptomes of invasive species can be rapidly sequenced, providing a resource to the research community without extensive breeding to create homozygous lines (e.g. generating 8 generations of an isofemale line). Such sequencing can provide a rapid response to pest invasions similar to that already described for epidemiology of infectious disease (e.g. [15]) and jumpstart molecular biology and genetic-based studies.

## Results

### Sequencing results

**Figure 1:**
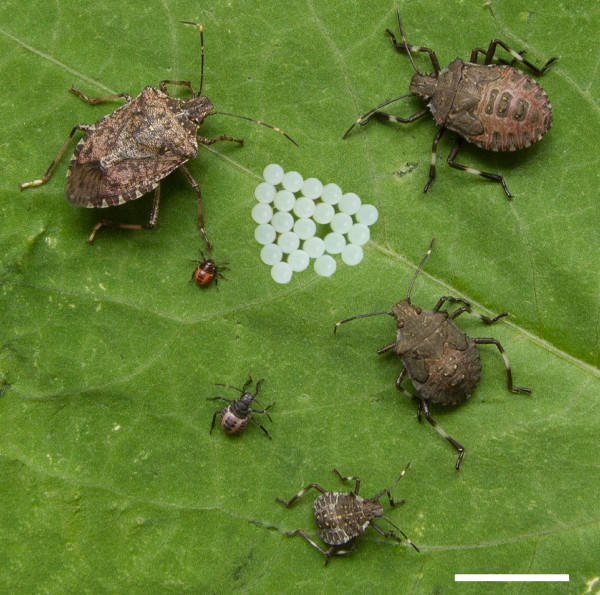
**Life stages of BMSB.** The life stages of BMSB are shown starting with eggs followed by 1^st^ instar nymphs, 2^nd^ instar nymphs, 3^rd^ instar nymphs, 4^th^ instar nymphs, 5^th^ instar nymphs, and an adult in a counter-clockwise spiral outwards and from largest to smallest. The bar in the low left represents 1 cm.

Whole transcriptome sequencing was undertaken in order to obtain gene sequences and jumpstart molecular biology studies focused on BMSB. RNA was collected for sequencing from 10 different stages and conditions including: (a) eggs, (b) 1^st^ instar nymphs, (c) 2^nd^ instar nymphs, (d) 3^rd^ instar nymphs, (e) 4^th^ instar nymphs, (f) 5^th^ instar nymphs, (g) an active adult male, (h) an active adult female, (i) an adult male in diapause, and (j) an adult female in diapause (Figure 1). Two pools were created with equimolar amounts of each RNA sample with pool A containing all of the pre-adult stages and pool B containing the adults.

**Figure 2:**
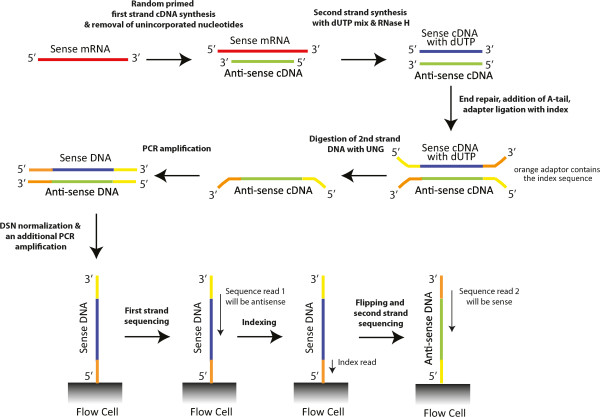
**Strand-specific sequencing with DSN normalization.** In strand-specific sequencing, the final sequencing reads can be assigned to a particular strand of DNA. This is accomplished by a second-strand synthesis with dUTP, ligation of adaptors to double-stranded DNA, and degradation of the second strand with UNG. The result is a fragment to be sequenced that is differentially labeled on the two ends in a manner that dictates how they are loaded on the sequencer and thus the order in which the ends are sequenced.

Each pool was used to generate a strand-specific library that was subsequently normalized with DSN (Figure 2). Strand-specific sequencing allows for assignment of the strand of transcription while normalization decreases the sequencing of the most abundant transcripts allowing for sequencing of more rare variants. Sequencing of these libraries on the Illumina HiSeq2000 resulted in 196,233,912 reads totaling >19 billion bases for Pool A and 170,455,294 reads totaling >17 billion bases for pool B. All of the reads were pooled and assembled with Trinity [16], which uses Inchworm to produce contigs via greedy k-mer extension, Chrysalis to produce components containing related contigs, and Butterfly to generate transcripts using the various de Bruijn graph paths in the components. We will refer to the assemblage of sequences generated by Butterfly as putative transcripts, given that some of the sequences that Butterfly assembles are not actually transcripts, as described below.

**Figure 3:**
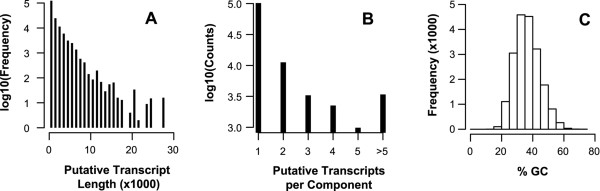
**Features of the trinity assembly. (A)** The size distribution of the putative transcripts on a logarithmic scale reveals that the vast majority of assembled transcripts are <1 kbp but transcripts >25 kbp can be assembled. **(B)** For the vast majority of genes there is only a single transcript assembled. **(C)** The % GC content of the putative transcripts peaks around 35%.

The resulting assembly contained 194,729 putative transcripts with 50,599 that were >1,000 bp and 20,076 having a top BLASTX match (e ≤ 10^−10^ and over >80% of the reference) to 8,354 unique Uniref90 protein sequences. For comparison, Trinity produced 196,000 putative transcripts with 14,522 that were >1,000 bp with 4,323 having a top BLASTX match (e ≤ 10^−10^) to 2,880 unique Uniref90 protein sequences over >80% of the reference protein length for an outbred *Bemisia tabaci* white fly population (Insecta: Hemiptera: Aleyrodidae) [17]. The longest assembled putative transcript from BMSB is 30 kbp in length; most components contained a single putative transcript; and the distribution of the %GC peaked around 37% (Figure 3).

### Strand-specificity and coverage

One problem with assigning a function to transcripts is determining which open reading frame is the correct one when there are open reading frames on both strands that lack evidence of function. This can be particularly true for organisms where good reference annotation is lacking, like insects. Sequencing a strand-specific library should assist in identifying the transcribed strand (Figure 2), reducing the number of possible ORFs by half. However, strand-specific libraries can have leakage, where experimentally the wrong strand is sequenced, that needs to be addressed.

**Figure 4:**
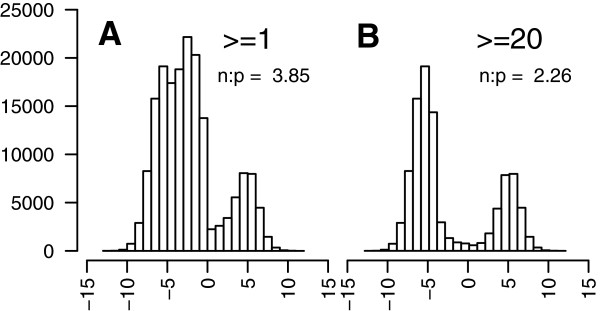
**Assessment of the strand-specificity of the RNA-Seq library.** The strand specificity of the sequencing library was based on comparing the number of first-in-pair reads mapped to the plus strand, to the corresponding number of second-in-pair reads. In a strand-specific library, the strand-specific log ratio (SSLR) should be away from zero. In the protocol used in this study, where the second-in-pair read comes from the sense strand, a negative SSLR is expected. **(A)** The distribution of SSLRs for putative transcripts having at least one proper read pair mapping to them demonstrates a preference for those with a negative SSLR. **(B)** When the plot is limited to those with twenty read pairs mapping to them, the bimodal distribution becomes more distinct, likely from the removal of contigs arising from contaminating genomic DNA.

To measure strand specificity, we calculated the log ratio of sense reads to antisense reads using Bowtie2 mappings that allowed for multiple matches, which will be referred to as the SSLR for strand-specific log ratio. We were able to show that overall the library is strand-specific with the plus strand preferred to the minus strand 3.40:1 (Figure 4A). While the SSLR demonstrates the plus strand was preferred, there was a significant amount of leakage that results in two putative transcripts that are reverse complements to one another. Leakage transcripts were identified as those that (a) matched another putative transcript in the alternate orientation using BLASTN, and (b) had a positive SSLR as described above. Using this approach, we discarded 32,308 putative transcripts originating from 24,507 components. Of those putative transcripts, there were 20,795 that had an ORF predicted on only one of the strands, and thus in only one direction. Not unexpectedly, 20,350 had their ORFs predicted on the minus strand compared to only 445 that had their ORFs in the plus strand. Overall, those with a SSLR < −5 had >10-fold increase in ORFs on the plus strand relative to the minus strand and those with a SSLR >5 had >10-fold increase in ORFs on the minus strand relative to the plus strand (Additional file 1: Figure S1). This indicates that using a SSLR can correctly predict the right strand.

**Figure 5:**
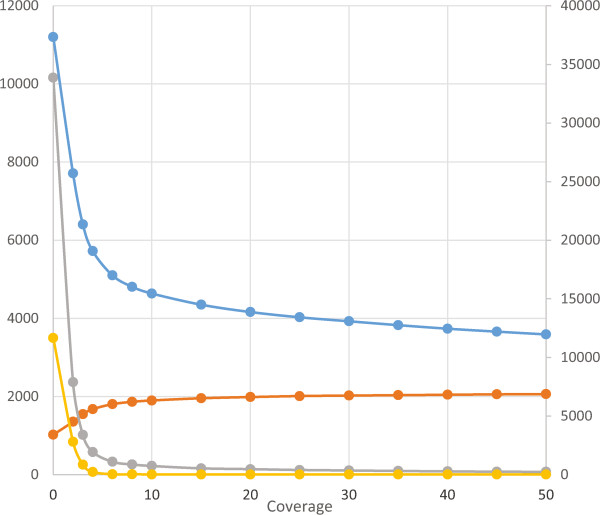
**Transcript properties relative to transcript coverage.** The mean length (orange), number of putative transcripts <300 bp (gray), and homology to an *E. coli* sequence (yellow) were plotted on the left axis while the number of putative transcripts (blue) were plotted on the right axis after applying a coverage threshold to the transcript (x-axis). A coverage cutoff of 20 maximizes the mean length and minimizes the number of transcripts with homology to *E. coli*.

Upon mapping the distribution of putative transcripts by strand specificity, one observes a bimodal distribution. The bimodal distribution becomes more symmetrical upon applying a coverage threshold. In particular, there are numerous putative transcripts with a strand specificity slightly less than zero that disappeared completely as more coverage was required to support the underlying putative transcript (Figure 4, Additional file 2: Figure S2). One explanation for this would be that these putative transcripts arise from contaminating genomic DNA that lacks strand specificity. This would manifest as short contigs supported by few reads and little strand specificity. Consistent with genomic DNA being present in the samples, applying a coverage cutoff of only two removed >10,000 putative transcripts that are <300 bp. In other projects, we have observed contaminating *E. coli* DNA that we suspect arrives in the sample through molecular biology reagents that may allow for an examination of our ability to detect genomic DNA contamination. In BMSB, bacterial DNA may also come from the microbiome, for example from an endosymbiont. However, regardless of the source, *E. coli* DNA could serve as a proxy for identifying genomic DNA in this sample*.* A coverage cutoff of two removed 3,501 putative transcripts that matched *E. coli.* If we plot the mean length, number of putative transcripts of size <300 bp, and number of reads with a match to *E. coli,* the greatest improvements are obtained with a coverage cutoff of 20 (Figure 5). Three putative transcripts still persist with homology to *E. coli.* One of these (comp3244_c38_seq1) has little strand specificity (SSLR = −0.45) and matches PhiX, which is added to Illumina sequencing runs and is not always completely removed through standard filtering. The remaining two, comp1098_c0_seq1 and comp1100_c0_seq1, are strand-specific, unique, and have BLASTN matches to the 16S and 23S rRNA, respectively, with best matches to the genome of an endosymbiont of the BMSB (GenBank: AP012554). This highlights that combining the results of the SSLR, a coverage threshold, and the BLASTN search significantly improves the transcriptome assembly and aids in identifying transcripts from both the host and its endosymbiont(s). The SSLR would be an easy and useful addition to implement into Trinity and other assemblers that would provide the greatest gains.

### Filtering to a single gene per component

While Trinity can generate almost 200,000 putative transcripts in >100,000 components, there are thought to be only 20,000-40,000 genes in Metazoan genomes. Applying the thresholds above reduces this to 53,071 putative transcripts from 18,573 components. However, analyses related to function, particularly those that result in statistical analyses, need the data to be reduced to a representative number of genes, thereby removing splice variants. Using an integrated approach, we were able to score each putative transcript based on two types of evidence including similarity to known sequences and the intrinsic features of each contig. We calculated a score for each of the remaining 53,071 putative transcripts of the Trinity assembly based on (a) similarity to known sequences, (b) features of any predicted ORFs, and (c) sequencing coverage along the putative transcript. The exact metrics used for calculating the score are described in Table 1 and Methods. This filtering resulted in 13,211 putative transcripts representing a 14-fold reduction in the number of putative transcripts and a 9-fold reduction in the number of components (Table 2). This number of putative transcripts is consistent with sequenced hemipteran genomes such as the 34,604 predicted genes in *A. pisum* [18].

**Table 1:** **Variables and their weights used to filter putative transcripts**

Attribute	Weight	Variable	Reason
**A) Based on the putative transcript sequence**			
1. What proportion of the database protein is covered in the first Uniref100 hit?	10	Proportion covered	Reward the putative transcript based on the proportion of the database protein that is covered in the best BLASTX hit
2. What proportion of the putative transcript is covered by the first Uniref100 hit?	8	Proportion covered	Reward the putative transcript based on the proportion of the query putative transcript that is covered in the best BLASTX hit
3. What is the length covered on the database protein in the first Uniref100 hit?	7	Database hit length/longest database hit length	Reward based on the absolute database protein length covered in the best BLASTX hit, compared to the longest hit length in the component
4. What is the length covered on the putative transcript in the first Uniref100 hit?	5	Putative transcript hit length/longest putative transcript hit length	Reward based on the absolute query putative transcript length covered in the best BLASTX hit, compared to the longest hit length in the component
5. Is the strand of the Uniref100 match, the expected one (based on SSLR)?	4	Match strand* (−SSLR/max |SSLR|)	Reward matches in plus strand if SSLR <0 or matches in minus strand if SSLR >0. In contrast, penalize matches in plus strand if SSLR >0 or matches in minus strand if SSLR <0
6. What proportion of the database protein is covered in the first NR hit?	9	Proportion covered	Same as the corresponding metric for Uniref100
7. What proportion of the putative transcript is covered by the best NR hit?	7	Proportion covered	Same as the corresponding metric for Uniref100
8. What is the relative length covered on the database protein in the first NR hit?	6	Database hit length/longest database hit length	Same as the corresponding metric for Uniref100
9. What is the relative length covered on the Trinity putative transcript in the first NR hit?	4	Putative transcript hit length/longest putative transcript hit length	Same as the corresponding metric for Uniref100
10. Is the strand of the NR match, the expected one (based on SSLR)?	3	Match strand* (−SSLR/max |SSLR|)	Same as the corresponding metric for Uniref100
11. Is the SSLR negative (i.e. the expected)?	7	- SSLR / max |SSLR|	Reward putative transcripts with the normal, negative SSLR
12. How long is the putative transcript compared to the longest in the component?	7	Putative transcript length/longest putative transcript length	Reward longer putative transcripts
**B) Based on the ORFs**			
1. Is the best match for each ORF the same?	10	(1 - Number of best matches)/number of best matches	Penalize putative transcripts having ORFs that have different hits.
2. Are there ORFs in both strands with both having an NR hit?	10	- Number of ORFs in strand "A"/number of ORFs in strand "B"	Maximum penalty if both ORFs have a NR hit
3. Are there ORFs in both strands with only one having an NR hit?	8	- Number of ORFs in strand "A"/number of ORFs in strand "B"	Intermediate penalty if only one of the ORFs has a NR hit
4. Are there ORFs in both strands with none of the two having an NR hit?	3	- Number of ORFs in strand "A"/number of ORFs in strand "B"	Small penalty if none of the ORFs have a NR hit
5. How many ORFs are called?	8	(1 - number of ORFs)/number of ORFs	Penalize putative transcripts having >1 ORFs
6. Are the ORFs found only in the expected strand (SSLR)?	8	ORF strand* (−SSLR/max |SSLR|)	Reward putative transcripts having ORFs called in only the expected strand
**C) Sequencing coverage dips**			
1. How many sequencing coverage dips?	10	- Number of dips/max number of dips in the component	Penalize putative transcripts with sequencing coverage dips

**Table 2:** **Summary of assembly and annotation**

Characteristic	Before filtering	After filtering
Number of reads (both pools)	366,689,206	N/A
Number of putative transcripts (both pools)	194,729	13,211
Average transcript length (bp)	1,005	2,026
Standard deviation (bp)	1,474	1,592
Median transcript length (bp)	439	1,649
Maximum transcript length (bp)	27,655	24,046
Transcripts >1000 bp	50,599	9,657
Transcripts with a Uniref100 hit (e-value < 1e-10)	80,536	11,513
Transcripts matching unique Uniref100 proteins	37,160	9,993
Transcripts with a NR hit (e-value < 1e-10)	80,262	11,497
Transcripts matching unique NR proteins	37,346	10,007
Number of Trinity components	123,175	13,211
Number of ORFs	89,684	13,210
Number of ORFs >450 bp	61,569	11,141
Number of ORFs with a function assigned	57,197	9,811

### COG Classification

**Figure 6:**
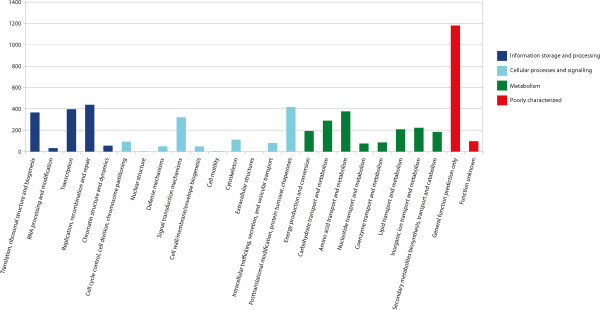
**Transcript functional categories.** The NCBI Cluster of Orthologous Groups (COG) database was used to classify the predicted proteins in the 13,211 representative transcripts. Assignment of COG categories showed that a large number of ORFs belonged to categories of proteins whose functions are poorly characterized, namely those that have general function prediction only and those with unknown function.

The NCBI Cluster of Orthologous Groups (COG) database was used to classify the predicted proteins in these 13,211 transcripts. Only 5,315 functions were assigned using NCBI COGs. Of those, almost a quarter (1,277) are functions that are not well categorized, namely general functional prediction and unknown function (Figure 6). The remaining categorizations are almost evenly divided between the other three categories of (a) information storage and processing, (b) cellular processes and signaling, and (c) metabolism. The top five sub-categories were (a) general function prediction only (22.2%); (b) replication, recombination and repair (8.2%); (c) posttranslational modification, protein turnover, and chaperones (7.9%); (d) transcription (7.5%); and (e) amino acid transport and metabolism (7.1%). The three categories comprising (a) extracellular structures, (b) nuclear structure, and (c) cell motility were the least abundant COGs represented, with 0, 2, and 4 matches (0.0%, <0.1%, and 0.1%), respectively. This is very similar to the distribution of COGs in other transcriptomes of eukaryotes, including insects [19–23].

### Polymorphisms

The laboratory colony used to generate RNA for transcriptome sequencing was established recently from field-caught BMSB. While there was at least one bottleneck in founding the colony, the assembled sequences were expected to have heterogeneity that reflects some of the genetic heterogeneity of the US BMSB population. Of the 26.6 million positions in the 13,211 transcripts that were examined with MPILEUP, 23.7 million positions were supported by >20 reads when all reads were aligned with Bowtie2. Of those positions, 212,422 had a substitution mutation that differed from the consensus base call that was supported by >5% of the underlying reads, which represents ~1% of the positions. If more stringent requirements are applied, 109,920 of these positions had >20% of the underlying reads with a substitution mutation. These positions were identified in 11,462 different putative transcripts, which is 86.8% of the total number of putative transcripts. The genetic heterogeneity observed may be significantly less than that found in the native Asian population, since the founder population in the US was likely small, representing a significant bottleneck, and an additional bottleneck occurred when founding the laboratory population. However, these polymorphisms may be a useful resource for population studies examining the spread of BMSB in the US.

### Differential expression between adults and non-adults

In order to compare the expression of juveniles and adults, the normalized log_2_Ratio of the read count was calculated between the two pools (Additional file 3: Table S1). A total of 22,707 putative transcripts had more than a 2-fold change in ratio, and 6,590 had more than a 16-fold change in ratio. The most highly expressed genes in the adult pool B that have functional annotation were vitellogenin genes, which is expected given that they encode the yolk protein produced in adult females. Similar levels of differential expression were found in a large number of hypothetical proteins and a protein with homology to an ankyrin protein found in bacterial endosymbiont *Wolbachia* strains. In the juvenile stages, the most abundantly expressed genes were the larval cuticle proteins, which is also expected. This, combined with the qRT-PCR validation of specific transcripts of interest described below, demonstrates that DSN normalized libraries can be used for a gene expression analysis that successfully identifies genes to be targeted for further characterization with other methods. The dynamic range of the expression changes is expected to be muted by the normalization procedure, and this is demonstrated for specific transcripts below.

Juvenile BMSB are more susceptible to insecticides than adults ([24, 25], and G. Dively, personal communication). Therefore, we sought to identify transcripts with functions related to pesticide detoxification that were up-regulated in BMSB adults. There were 25 genes encoding proteins related to cytochrome P450 and glutathione S-transferase that were more abundant in adults (Table 3). There were nine such proteins that were more abundant in the pre-adult RNA pool (Table 3).

**Table 3:** **Differentially expressed detoxification genes**

Name	Functional annotation	Stage up-regulated
Comp25785_c0_seq1	Cytochrome P450 gene	Adults
Comp26191_c0_seq1	Glutathione S-transferase	Adults
Comp15122_c0_seq1	Cytochrome P450 gene	Adults
Comp16607_c1_seq2	Cytochrome P450 gene	Adults
Comp15049_c0_seq1	Cytochrome P450 gene	Adults
Comp15912_c1_seq2	Cytochrome P450 gene	Adults
Comp20672_c0_seq4	Cytochrome P450 gene	Adults
Comp13685_c1_seq2	Cytochrome P450 gene	Adults
Comp40610_c0_seq2	Cytochrome P450 gene	Adults
Comp8954_c0_seq1	Glutathione S-transferase	Adults
Comp20241_c0_seq1	Cytochrome P450 gene	Adults
Comp18070_c0_seq2	Cytochrome P450 (2 ORFs)	Adults
Comp11443_c0_seq1	Cytochrome P450 gene	Adults
Comp18881_c0_seq1	Cytochrome P450 gene	Adults
Comp7095_c0_seq2	Cytochrome P450 gene	Adults
Comp14891_c0_seq2	Cytochrome P450 gene	Adults
Comp8170_c0_seq1	Cytochrome P450 gene	Adults
Comp4236_c0_seq1	Probable cytochrome P450 gene	Adults
Comp2339_c0_seq1	Cytochrome P450 gene	Adults
Comp6322_c0_seq2	Cytochrome P450 gene	Adults
Comp17381_c0_seq6	Cytochrome P450 gene	Adults
Comp23582_c1_seq1	Cytochrome P450 gene	Adults
Comp4238_c0_seq1	Cytochrome P450 gene	Adults
Comp3991_c0_seq3	Glutathione peroxidase (3 ORFs)	Adults
Comp8540_c0_seq1	Probable cytochrome P450 gene	Adults
Comp6146_c0_seq1	Glutathione S-transferase	Pre-adults
Comp11026_c2_seq1	Cytochrome P450 gene	Pre-adults
Comp21713_c0_seq1	Cytochrome P450 gene	Pre-adults
Comp3892_c0_seq1	Cytochrome P450 gene	Pre-adults
Comp18921_c0_seq3	Catalase	Pre-adults
Comp25932_c0_seq1	Cytochrome P450 gene	Pre-adults
Comp10873_c0_seq1	Probable cytochrome P450 gene	Pre-adults
Comp12303_c0_seq1	Cytochrome P450 gene	Pre-adults
Comp8344_c0_seq1	Probable cytochrome P450 gene	Pre-adults

### Taxonomic profile

The putative transcripts were searched against NR using BLASTX and a lowest common ancestor (LCA) assignment [26] was made by aggregating all of the matches with the best e-value. Prior to filtering the putative transcripts to remove ones we suspected arose from genomic DNA as described above, only 78,489 (89%) putative transcripts had a eukaryotic LCA of a total of 88,630 putative transcripts with BLAST results (Additional file 4: Figure S3). Of the 7,111 (8%) putative transcripts with a bacterial LCA, 6,366 had an LCA to γ-Proteobacteria with *Escherichia coli* as the most common species level assignment (Additional file 5: Figure S4)*.* Following filtering, 11,359 (96%) putative transcripts had a eukaryotic LCA of a total of 11,854 putative transcripts with a BLAST result (Additional file 6: Figure S5). Of the 120 (1%) putative transcripts with a bacterial LCA, only 18 had an LCA to γ-Proteobacteria, and *E. coli* was no longer the most common species level assignment (Additional file 7: Figure S6)*.* This suggests that the SSLR-based filtering described in the prior section was effective at removing contaminating genomic DNA in the library.

Intrigued by the presence of a transcriptionally regulated gene of *Wolbachia* ancestry in the BSMB transcriptome, we sought to investigate the ancestry of these bacterial transcripts further. The remaining bacterial putative transcripts have homology to numerous endosymbionts. LCAs for the endosymbiont of the brown-winged green stink bug *Plautia stali* [27] and *Pantoea* spp. were detected, suggesting relatives of these bacteria may be present as endosymbionts in this population of BMSB. This is consistent with the recent identification of *Pantoea agglomerans* as a BMSB endosymbiont [28]. The highest number of bacterial LCAs (53 putative transcripts or 44%) were from *Amoebophilus asiaticus* in the Bacteroidetes, which is an endosymbiont of free-living amoebae [29]. *A. asiaticus* is related to insect endosymbionts including *Blattabacterium* spp. in cockroaches [30], *Sulcia muelleri* in cicada [31], and *Cardinium hertigii* in parasitoid wasps [32]. While the BLASTX returned an LCA of *Amoebophilus,* the BLASTN searches did not return any significant matches for any of these, indicating a high level of divergence for the nucleotide sequences. Most of the proteins with a bacterial LCA had homology to ankyrin repeat containing proteins found in diverse endosymbiont and invertebrate genomes, including all of the proteins with an *Amoebophilus* LCA. Additional transcripts with bacterial LCAs encode proteins with homology to mannanases, amylase, C4-dicarboxylate transporter, and three different hypothetical proteins.

### Ankyrin repeat containing proteins with significant bacterial homology

There were 29 LCAs to α-Proteobacteria, all of which were in the Rickettsiales, including 27 that were to *Wolbachia* endosymbionts. Since *Wolbachia* endosymbionts are found in many insects, we tested for the presence of *Wolbachia* using a robust set of 40 primers that collectively amplify genes from a diverse array of *Wolbachia* endosymbionts as well as other microorganisms in the family Anaplasmataceae [33]. We did not detect any amplification products for *Wolbachia* endosymbionts using BMSB genomic DNA, while the primers amplified products using control DNA. This suggests that this BMSB colony is not colonized by a *Wolbachia* endosymbiont.

Three putative transcripts were identified that were differentially expressed and had homology to *Wolbachia* ankyrin proteins including comp549_c15_seq1, comp2753_c5_seq1, and comp18511_c0_seq1, which had SSLRs of −4.8, −5.4, and −6.2, respectively. Differential expression analysis of the transcriptome data reveals 28-fold and 776-fold overexpression of comp549_c15_seq1 and comp2753_c5_seq1 in the adult pool while comp18511_c0_seq1 was 104-fold overexpressed in the juvenile pool. These results were confirmed by qRT-PCR using the original RNA samples, prior to pooling. For qRT-PCR we examined comp549_c0_seq3, instead of comp549_c0_seq1, as it is slightly longer and contains part of the latrotoxin domain discussed below. These qRT-PCR experiments demonstrate a specific 20,000-fold and 17,000-fold increase in expression of comp549_c15_seq3 and comp2753_c5_seq1 in active adult females when compared to all other stages, and a specific 170-fold increase in expression of comp18511_c0_seq1 in 5^th^ instar nymphs.

While all three putative transcripts have homology to known ankyrin-repeat containing proteins, comp2753_c5_seq1 and comp549_c15_seq3 do not contain ankyrin (ANK) repeats. Both have homology to a large multiple domain protein in *Wolbachia* endosymbionts of *Culex pipiens* (e.g., WP_007302981.1) that has a latrotoxin domain at its C-terminus and numerous ankyrin repeats in the N-terminal half. The homology for comp549_c15_seq3 spans a portion of the latrotoxin domain and the adjacent ANK repeats, while the homology for comp2753_c5_seq3 spans only part of this region. This region of homology is at the C-terminus of BMSB ORFs while the 560 and 223 amino acids at the N-terminus have little homology for comp549_c15_seq3 and comp2753_c5_seq1, respectively. No changes in sequencing coverage around these regions were identified in either transcript that would suggest a misassembly. The N-terminus of the ORF encoded in comp549_c15_seq3 contains a JNK_SAPK-associated protein-1 domain.

Examination of the phylogenetic relationship of comp549_c15_seq3 and comp2753_c5_seq1 with homologous sequences in GenBank (<e-15), reveals that while they have homology to *Wolbachia* endosymbionts (α-Proteobacteria), collectively they also have homology to coding sequences (CDSs) in *Rickettsiella grylii* (γ-Proteobacteria) and *Diplorickettsia massiliensis* (α-Proteobacteria) (Additional files 8 and 9: Figures S7-S8). The phylogenetic diversity of the bacterial taxa suggests that there have been lateral gene transfers involving diverse arthropod-associated bacterial lineages and the precise donors and recipients of these bacteria-bacteria lateral gene transfers are not clear. While we suspect that this may also be a lateral gene transfer involving BMSB, we cannot rule out the presence of these genes arising from an endosymbiont of BMSB. By qPCR, these genes were at the same relative abundance as BMSB nuclear genes, but we were not successful at placing these genes in the BMSB genome using inverse PCR. The ongoing BMSB genome sequencing project will likely clarify this issue. Should this prove to be a lateral gene transfer, it is not clear whether the gene moved from bacteria into insects, or vice versa.

The third transcript (comp18511_c0_seq1) contains an ORF with full length matches to *Wolbachia* proteins that contain ANK repeats and a PRANC domain. PRANC domains were at one time described only in Pox viruses, where PRANC-containing proteins inhibit NF-κB activation by TNF-α, thereby altering the innate immune response of the mammalian host. More recently PRANC domains were identified in the human pathogen *Orientia tsutsugamushi* (Bacteria: Rickettsiales), multiple *Wolbachia* endosymbionts (Bacteria: Rickettsiales), *Wolbachia* phage, and *Nasonia vitripennis* (Eukaryota: Hymenoptera). Previous phylogenetic analysis suggests that the *Nasonia* lineage acquired one or more of these proteins from a *Wolbachia* endosymbiont via lateral gene transfer [34]. Examination of the phylogenetic relationship of the BMSB sequence with homologous sequences in GenBank (<e-15), reveals that while it has homology to *Wolbachia* endosymbionts (α-Proteobacteria), it is distinct from the *Wolbachia* proteins. It is not clear if the BMSB protein represents an ANK-PRANC protein in another bacterial lineage or arthropod lineage. As with the genes described above, qPCR results revealed the same relative abundance as BMSB nuclear genes and is consistent with these genes being in the BMSB nuclear genome. However, in both cases it is possible an endosymbiont is present whose genome also has the same relative abundance as the BMSB genome.

### Sugar polysaccharide metabolism proteins with bacterial homology

The next most abundant proteins with homology to bacterial proteins were those encoding mannanases and amylases. These genes are of particular interest as they could aid in the degradation of plant cell wall polymers and storage molecules.

While the amylase transcripts had best homology to bacterial genes in NR, they had very good homology to transcripts from other diverse insect genera including *Drosophila, Nasonia, Aedes, Anopheles, Rhodnius,* and *Apis.* A phylogenetic analysis had poor bootstrap support for most branches and thus did not adequately resolve the amylase genes. The results are therefore inconclusive, but most consistent with vertical inheritance of these genes within insects. The copy number in genomic DNA, as assessed by qPCR, is consistent with the presence of the amylase gene in the host chromosome and its qRT-PCR results are consistent with it being constitutively expressed from the insect nuclear genome.

**Figure 7:**
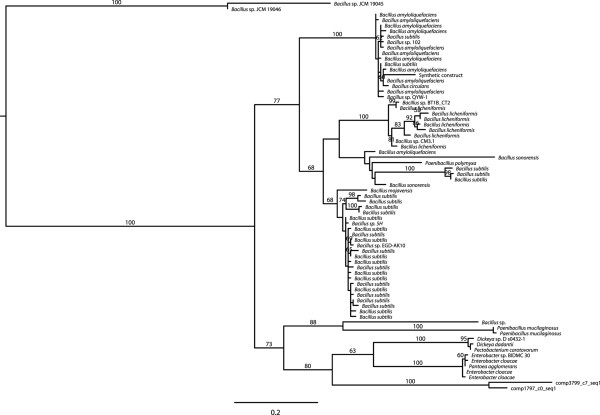
**Mannanase C-terminus phylogeny.** Proteins that were homologous to the C-terminus of the putative mannanase proteins were identified using a BLASTX [35] search of NR, aligned with CLUSTALW [36], and maximum likelihood phylogenies generated using RAxML [37]. This reveals the presence of the BMSB mannanase in a well-supported clade of bacterial homologues from bacteria in the genera *Dickeya Pectobacterium, Enterobacter,* and *Pantoea*.

The mannanase genes, however, showed no homology in NR to any insect genes. A complete mannanase transcript was not assembled. Instead, the assembly includes multiple fragments for the 5′-end and the 3′-end of the gene that may constitute 3–4 complete mannanase genes. PCR was used to demonstrate that comp17117_c0_seq1 and comp14839_c0_seq1 were part of a larger transcript that would encode a complete mannanase as would comp2467_c6_seq1 and comp1797_c0_seq1. Phylogenetic analysis of all of the overlapping 3′-fragments showed strong support for a BMSB clade that was sister to one containing bacteria from the genera *Dickeya*, *Pectobacterium*, *Enterobacter*, and *Pantoea*, the latter of which is the genus designation for at least one BMSB endosymbiont [28] (Figure 7). Phylogenetic analysis of the 5′-fragments showed strong support for a BMSB clade but did not resolve its position with respect to the bacterial sequences (Additional file 10: Figure S9).

Three mannanase fragments (comp2467_c6_seq1, comp7015_c0_seq1, and comp11444_c0_seq1) were selected for further examination by qRT-PCR and qPCR since the former was found to be up-regulated in pre-adults in the differential expression analysis, while the latter two were found to be up-regulated in adults. By qRT-PCR, comp2467_c6_seq1 clustered with constitutively expressed nuclear genes and had Ct values similar to BMSB nuclear genes. Comp7015_c0_seq1 and comp11444_c0_seq1 were poorly expressed in all stages consistent with transcription from the endosymbiont genome, but had their highest levels of expression in active females.

All of the mannanase and non-mannanase primer pairs designed for qRT-PCR and qPCR experiments generated an appropriately sized amplicon on RNA and generated no amplicon when reverse transcriptase was omitted. However, numerous of those primer pairs failed to produce the same amplicon on DNA. In the case of the L27 control primer pair, we suspect the primers may span an intron; as such they fail in qPCR but perform well for qRT-PCR. An intron may also be present for one of the mannanase genes (comp2467_c6_seq1) where the wrong size fragment was observed with genomic DNA. In contrast to L27, the comp2467_c6_seq1 primers still resulted in good Ct values in the qPCR and qRT-PCR. The qPCR Ct values are similar to those for genes in the BMSB chromosome, so we suspect that this mannanase gene is in the BMSB genome. However, two other mannanase primer pairs (comp7015_c0_seq1 and comp11444_c0_seq1), and all of the control primer pairs targeting endosymbiont transcripts, failed to produce an amplicon or valid qPCR results. As such, we suspect that the corresponding mannanase genes are found in an endosymbiont genome. We could not identify it in the publicly available endosymbiont genome (GenBank: AP012554), but it is possible that there are multiple endosymbionts. Taken together, this suggests that related mannanase genes are present in both BMSB and its endosymbiont(s) and that both are abundant enough to be detected in this experiment. This suggests that mannanase may play a significant role in BMSB. All of the genes ascribed to the endosymbiont based on qPCR results had good Ct values in qRT-PCR experiments with RNA from active females. This may indicate that the active female examined here had a robust endosymbiont transcriptional response.

### Bacteria-to-hemiptera lateral gene transfer

Previously, 12 genes or gene fragments have been identified in the pea aphid genome that resulted from LGT from bacteria [38, 39]. Given that BMSB and aphids are both Hemiptera, we sought to investigate if these genes were also present in the BMSB transcriptome. Two transcripts (comp4381_c3_seq1 and comp4662_c1_seq1) were identified that encode bacterial lysozymes, or bLys proteins. Both showed good support with 9,337 and 8,081 reads, respectively, and strand specificity with SSLRs of −5.5 and −4.4, respectively.

**Figure 8:**
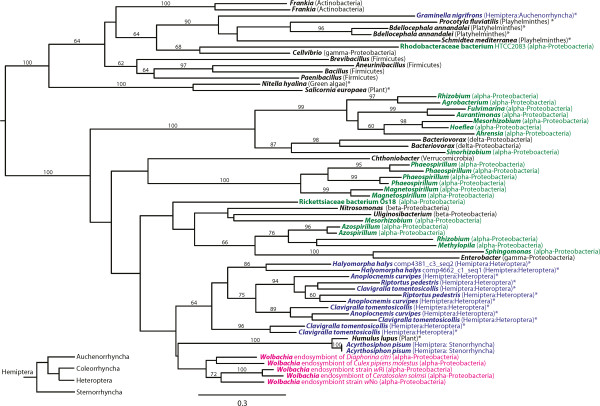
**Lysozyme phylogeny.** Homologues to the two putative lysozymes from BMSB were identified using a BLASTX [35] search of NR and the NCBI whole transcriptome database, aligned with MAFFT [40], and maximum likelihood phylogenies generated with RAxML [37]. The two lysozymes cluster with lysozymes from other Hemiptera as well as α-Proteobacteria. This cluster includes the lysozymes that have previously been attributed to lateral gene transfer from bacteria in the order Rickettsiales, which includes *Wolbachia* endosymbionts of arthropods. Presence in multiple hemipteran lineages may suggest that this transfer predates the divergence of all Hemiptera. Outside the eukaryotic and *Wolbachia* clades, only the genus is provided, in order to aid visualization. Sequences taken from whole transcriptome assemblies are denoted with asterisks since it is important to carefully consider that the sequences may have arisen from contamination from the microbiome. The inset in the lower left corner illustrates the phylogeny of the suborders of Hemiptera [41].

These transcripts have homology to numerous other hemipteran lysozyme genes that have been attributed to LGT from a bacteria related to *Wolbachia* endosymbionts. The hemipteran lysozyme transcripts are not well supported in the phylogeny of lysozymes with less than 70% bootstrap support (Figure 8). However, the heteropteran lineage transfers are monophyletic, albeit not well supported. The heteropteran clade was sister to a clade consisting of Sternorrhyncha and *Wolbachia* endosymbionts and nested in a well-supported clade of lysozymes from α-Proteobacteria with 100% bootstrap support. This suggests an old lateral gene transfer that predates the divergence of large families of Hemiptera.

Therefore, we sought to establish the distribution of this lateral gene transfer in Hemiptera. It could be identified in the genome sequencing projects of *A. pisum* and *Riptortus pedestris*, the only Hemiptera whole genome sequences available at the time of this analysis. Using histone H3 (which is highly conserved and essential in insects) to query the transcriptome shotgun assemblies deposited at NCBI with TBLASTN, we were able to identify seven Hemiptera in the repository including *Rhodnius prolixus* (Heteroptera), *Aphis craccivora* (Sternorrhyncha), *Graminella nigrifrons* (Auchenorrhyncha), *Clavigralla tomentosicollis* (Heteroptera), *Triatoma matogrossensis* (Heteroptera), *Bemisia tabaci* (Sternorrhyncha), and *Anoplocnemis curripes* (Heteroptera). We were able to identify bLys in three of these seven transcriptome shotgun assemblies, including *G. nigrifrons, C. tomentosicollis,* and *A. curripes*. Absence of these genes in the other four insects could be due to many factors, including incomplete sampling of genes with transcriptome sequencing as well as removal of bacterial transcripts prior to deposition. As an example of the latter screening, the bLys proteins encoded in these transcriptome assemblies were missing from the corresponding protein database at NCBI when searched with BLASTX. Likewise, the contigs containing the bLys proteins in the aphid genome have been removed recently, despite numerous pieces of experimental evidence demonstrating their presence in the aphid genome.

However, the presence of bLys genes in all hemipteran genomes sequenced thus far, their presence in several transcriptomes including BMSB, their relative abundance as measured by qPCR, and their presence in a clade dominated by α-proteobacterial lysozymes suggests that these genes are not in an endosymbiont genome but instead in the hemipteran genome. This suggests the existence of one or more ancient transfers of bacterial lysozyme into this lineage. Both bLys transcripts contained only the bacterial 1,4-beta-N-acetylmuramidase, and not the eukaryotic peptidase that is fused to it in aphids. Neither of the BMSB bLys transcripts were differentially expressed between the adult and pre-adult RNA pools. Examination by qRT-PCR reveals constitutive expression across all of the life stages with transcript abundance levels similar to other constitutively expressed genes.

## Discussion

### Rapid sequencing of an insect pest

BMSB is a major agricultural pest of the mid-Atlantic region of the US and is one of the top priorities for control set forth by the USDA. Yet there has been a lack of sequence data to facilitate molecular biology studies of this species. One barrier to genome sequencing is the need to have a sizable inbred population in order to obtain the necessary amount of nucleic acid for sequencing while maintaining the homogeneity necessary to facilitate whole genome assembly. This is particularly true for complex eukaryotic genomes, for which large quantities of DNA are needed to generate either large insert libraries or long sequence reads. However, many organisms do not have established laboratory colonies, and for emerging pests, methods have to be developed *de novo* to maintain such colonies for the many generations needed to successfully generate an inbred population.

Transcriptome sequencing can fill the void in these instances by rapidly providing the sequence of much of the coding potential of the genome. Because coding sequences are under constraint, they tend to be less variable and, at least in this instance, seem to be reasonably well resolved without the time-consuming measures needed to create genetically homogenous lines. Rapid sequencing has gained a great deal of interest for bacteria in clinical settings (e.g.[15]). Clinical samples can be obtained, sequenced, and analyzed in less than a week’s time. In a similar manner, improvements to whole transcriptome sequencing for eukaryotes with larger genomes could aid in rapid development of resources for emerging ecological pests that threaten agriculture or the environment.

One might expect transcriptome sequencing to be simpler than whole genome sequencing given the short sequences to be assembled and the absence of introns. However, *de novo* transcriptome assembly and analysis can still prove to be quite challenging. One problem arises from the variable coverage between different transcripts. The other main challenge comes from alternative splicing and the sequencing of incompletely spliced transcripts. Alternative splicing can be observed in different tissues and at different developmental stages. As a result, a larger number of alternatively spliced mRNAs are expected from samples derived from multiple tissues and/or developmental stages. Coincidentally, transcriptome assemblies generally use data from a wide variety of tissues and stages in order to increase our ability to find more genes, which compounds this problem.

While knowing the splice variants in a transcriptome can be important, in some cases a single “representative” transcript of each gene is a necessary first step, in order to reduce complexity and facilitate downstream comparative analyses. One approach [23] is to take the longest transcript, with the rationale that it is probably the full-length transcript. However, we found long transcripts in this dataset that resulted from the erroneous joining of two smaller transcripts through misassembly, suggesting this is not always a good metric. Other studies have used filtering based only on BLAST results [42], or filtering based on only intrinsic properties of the assembly [43]. Our approach combined both intrinsic properties of the putative transcripts, as well as their similarity to sequence databases, in order to find the representative transcript from a group of related transcripts (Table 1) in Trinity components. The most important intrinsic properties include (a) sequencing coverage, (b) strand specificity, (c) length, (d) strand of predicted ORFs, and (e) sequencing coverage changes. Assessment of these properties results in a highly integrated pipeline for filtering the list of putative transcripts generated by Trinity that examines multiple dimensions, giving different user-specified weights to properties of different biological importance to best address the researcher’s biological question. Applying the technique here revealed many novel observations about transcripts of bacterial ancestry.

### Mannanase

Several transcripts were identified with homology to mannanase genes. *Hypothenemus hampei* (Coleoptera) has acquired a mannanase gene from bacteria through lateral gene transfer that enables parasitism of coffee berries relative to sister taxa [44]. However, it has little sequence similarity to the putative mannanase genes discovered here. Prior to discovery of the mannanase gene in *H. hampei* through sequencing genes in the secretome, mannanase genes were not described in insects [44]. Therefore, the presence of mannanases in the BMSB and its endosymbiont is intriguing. Mannanase degrades mannan, a plant polysaccharide storage molecule composed primarily of mannose that is frequently found in seeds. BMSB has been a major pest on soybeans where they feed directly on individual soybean seeds after piercing through the pods, which results in flattened pods [45]. As such, mannanase could be an important addition to the metabolic repertoire of BMSB and its endosymbiont, leaving them well-adapted to feed on soybeans. This also suggests that mannanase may be a good target for RNAi-mediated control of BMSB. The relative abundance of the mannanase genes measured suggests multiple genomes contribute mannanase genes. The results are most consistent with the presence of mannanase in one or more endosymbionts and the BMSB nuclear genome. The *H. hampei* mannanase had little sequence similarity to the putative mannanase genes discovered here, suggesting that any lateral gene transfer would be an independent acquisition of these genes.

### Ankyrin repeat proteins

**Figure 9:**
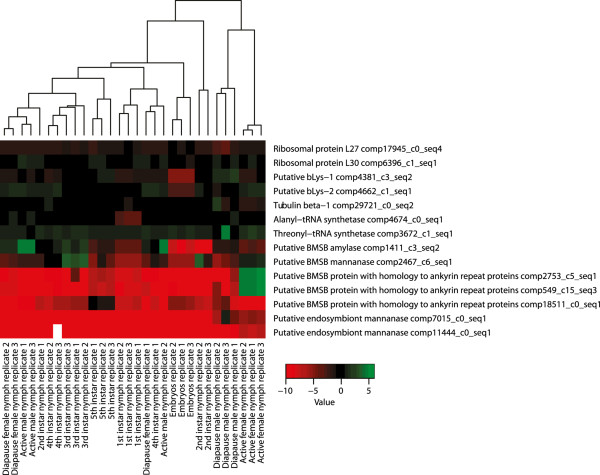
**Heat map of qRT-PCR data.** All ten individual RNA samples were subjected to qRT-PCR for five insect housekeeping genes and nine genes with best hits to bacterial genes using BLAST. Within each sample the ∆Ct is illustrated in a heat map and was calculated as the difference between the average Ct across the four constitutively expressed genes (ThRS, L30, Tubulin, and L27) and the Ct value for the gene being interrogated. In this way the color coding represents the difference in expression between the gene queried and the average constitutively expressed housekeeping gene, which all had similar abundance levels. In most samples, comp18511_c0_seq1, comp549_c15_seq3, comp2753_c5_seq1, comp7015_c0_seq1, and comp11444_c0_seq1 were poorly expressed relative to the constitutively expressed genes. However, in fifth instar nymphs (dark green), comp18511_c0_seq1 was transcribed at levels similar to the constitutively expressed genes while in active adult females, comp549_c15_seq3 and comp2753_c5_seq1 were transcribed at levels 64-fold higher than the average constitutively expressed gene. Both bLysB, the putative amylase, and the putative BMSB mannanase transcripts were transcribed constitutively at levels similar to the other constitutively expressed genes. For RNA that did not amplify a Ct value was assigned of 43, which was the lowest Ct value measured across the dataset. This reflects the low abundance of a transcript that did not amplify, but still allowed for clustering. The value for comp11444_c0_seq1 in the 4^th^ instar larvae replicate 3 is missing due to an aberrant amplification curve.

Two transcripts, comp2753_c5_seq1 and comp549_c15_seq3, were identified as having strong similarity to a specific ankyrin repeat protein of *Wolbachia* endosymbionts that contains a C-terminal latrotoxin C domain. Latrotoxin is best known for being the active component in black widow spider venom, but a homologue has also been identified in *Wolbachia* endosymbionts and *Rickettsiella grylli* [46]. The BMSB transcripts have homology to these proteins in these organisms (Additional files 8 and 9: Figures S7-S8). However, they do not contain ankyrin repeats or the complete latrotoxin domain nor do they have similarity to any protein domain in the PFAM database. We demonstrate that these transcripts are up-regulated in adult females (Figure 9) and that they are most likely encoded in the BMSB nuclear genome.

These transcripts are intriguing given their differential and increased expression in BMSB and the interesting functions of their homologues in other insects. For example, the salivary gland secreted (SGS) family of proteins in mosquitoes are in the same family of proteins [46]. SGS proteins are specifically transcribed in the salivary glands, localized to the basal lamina, and are necessary for successful invasion of the mosquito salivary glands by sporozoites of the protozoan parasite *Plasmodium gallinaceum* [47]. In SGS proteins, the latrotoxin domain is replaced by a Tox-SGS domain that is further composed of heparin binding domains [46, 47]. Given their limited distribution among arthropods, it has been hypothesized that both the spider latrotoxins and the mosquito SGS proteins were acquired from bacteria closely related to *Wolbachia* and were subsequently co-opted for arthropod-specific functions [46]. However, these protein families are also not widely distributed among bacteria, and thus far seem to only be associated with insect endosymbionts. Previously, analyses focusing on SGS proteins have supported LGT between bacteria and insects, although the directionality of the transfer was not clear [48, 49]. Neither the role nor the origin of these transcripts in BMSB is clear, but both warrant further investigation.

### Lysozyme

Given the diversity of Hemiptera genomes and transcriptomes that encode bacterial lysozyme, including representatives from Heteroptera and Sternorrhyncha, and given the clustering of these lysozyme genes with α-proteobacterial lysozymes, the lysozyme genes in Hemiptera likely result from one or more lateral gene transfer(s) that occurred prior to the radiation of this order or its suborders. This suggests an ancient transfer or transfers that we sought to investigate further.

In aphids, it was suggested that the bacterial lysozyme might compensate for the lack of the canonical c-type lysozyme found in metazoans, including insects [38]. However, sequencing revealed the presence of two transcripts from c-type lysozymes as well as a transcript from the bacterial lysozyme in *R. pedestris* [50]. BMSB have twenty putative transcripts with homology to 66 of the 67 *D. melanogaster* lysozyme proteins in NR and 7 of the 7 *D. melanogaster* lysozyme proteins in ImmunomeBase [51], suggesting that BMSB has a similar complement of lysozyme genes as *D. melanogaster*. This suggests that the presence of the bacterial lysozyme is not strictly related to the loss of the c-type lysozyme in all Hemiptera.

Hemiptera include many phloem-feeding insects including aphids and other pests, where economically important crop damage is related to the piercing of the plant tissue. Hemiptera have also received a great deal of attention for frequently containing primary mutualistic endosymbionts. These two traits are likely related since phloem is nutritionally incomplete and the primary mutualistic endosymbionts often provide the essential nutrients that the insect requires that are not provided by the phloem. Therefore, Hemiptera-specific differences relative to other insects may be associated with acquisition of such endosymbionts and a transition of these insects to this food source.

We hypothesize that to obtain primary endosymbionts Hemiptera may have had to relax their immune response to bacteria, relative to their ancestors. The lysozyme LGT could have played a role in this. Aphids are reported to lack homologues to many of the innate immune genes that target bacteria including peptidoglycan receptor proteins (PGRPs), the IMD signaling pathway, and antimicrobial peptides [52]. The BMSB transcriptome had 95 of the 97 identified aphid immune-related proteins using BLASTP (e < 10^−5^), but were lacking transcripts for Galectin 1 and Spätzle 6 using these criteria. Using a BLASTP search (e < 10^−5^) with *D. melanogaster* genes from ImmunomeBase [51], we were able to identify numerous immune-related genes in BMSB that were absent in the pea aphid including those encoding PGRPs, Eater, Traf6, Kayak, and Defensin. We speculate that with a complement of PGRPs, BMSB may be able to activate defenses against bacteria more readily than the pea aphid, although this would have to happen in the absence of a functional IMD signaling pathway.

However, this response is likely still not as robust as that of *D. melanogaster.* A TBLASTN search of the entire NCBI shotgun transcriptome database with the *D. melanogaster* homolog to *dFadd* (NP_651006.1) failed to identify a homolog in any Hemiptera despite allowing the search to return 5000 results, which yielded many more divergent metazoan homologs. This is consistent with widespread loss of IMD signaling in multiple Hemiptera. Analogous searches failed to identify *imd*, *Dredd*, or *kenny* homologs in any Hemiptera shotgun transcriptome except the *Gramminella* transcriptome. This is intriguing since the *Gramminella* bacterial lysozyme is the only one that does not cluster with the lysozymes from α-Proteobacteria. *Gramminella* is also the only representative of Auchenorrhyncha examined. Given the relationship of the Hemiptera suborders (Figure 8, inset), this raises the possibility that both the loss of the IMD pathway and acquisition of an α-proteobacterial lysozyme occurred independently in the Sternorrhyncha and Heteroptera lineages.

Acquisition of lysozyme and loss of IMD may be features that are common to many Hemiptera given this examination of the shotgun transcriptome database. It has been proposed that the loss of immune genes in the pea aphid may be compensated for by an equally effective alternate response [52], although the genes of this alternate response could not be identified by suppressive subtractive hybridization (SSH) upon challenge with a Gram-negative bacteria [53]. It was also proposed that “defensive” endosymbionts may be able to compensate for the loss of these functions [52]. However, this was noted as a chicken and egg problem since loss of these functions would be necessary for endosymbiont colonization [52]. We propose that acquisition of lysozyme provides broad-spectrum protection against a subset of bacteria (e.g. Gram-positive bacteria) facilitating loss of more general pathways that respond to all bacteria (e.g. IMD). Such an alteration would facilitate colonization by primary and secondary endosymbionts, most of which are Gram-negative Proteobacteria. A Gram-positive response would be consistent with the SSH experiments that were only conducted with a Gram-negative species. While intriguing, the hypothesis that acquisition of lysozyme enabled immune function loss and subsequently endosymbiont gain is only conjecture, but it could be the focus of future investigations. Of note, bLys could be a useful target for the development of insecticides, given its bacterial origin, potential role in defending against bacterial invasion, and its presence in this clade with numerous agriculturally significant pests.

## Conclusions

BMSB is a particularly difficult invasive agricultural pest to manage with limited treatment options and an ability to spread. Transcriptome sequencing was undertaken to provide a molecular resource to the research community for genetic- and genome-based research. The polymorphisms identified in this data set can be used to examine the population genetics of BMSB, which will allow for a better understanding of the original invasion in Allentown and its movement throughout the US and world. Understanding these invasions will increase our understanding of how to prevent the introduction of invasive pests in the future. Our study demonstrates that the transcriptomes of invasive species can be rapidly sequenced providing a resource to the research community without extensive breeding to create homozygous lines. Furthermore, strand-specific transcriptome sequencing can facilitate the identification of transcripts. Here, transcriptome sequencing enabled us to identify putative lateral gene transfers between bacteria and insects including ankyrin-repeat related proteins, lysozyme, and mannanase. These Hemiptera- and BMSB-specific genes provide novel and species-specific targets for the development of genome based methods to control invasive pests.

## Methods

### Insect husbandry and RNA isolation

Lab colonies of BMSB were maintained as previously described [28]). Briefly, wild-caught insects were collected in soybean fields at the University of Maryland Beltsville Research Farm. The insects were reared in mesh cages (60 × 30 × 35 cm) on potted plants of *Phaseolus vulgaris*, excised bean pods, and raw sunflower seeds at 25°C, RH of 65 ± 5%, and a 16 h light with 8 h dark photoperiod. RNA was extracted from wild-caught adults whereas all other life stages were collected from cages where wild-caught females had offspring. No purposeful efforts were taken to inbreed to create a homozygous line. All stages were flash frozen in liquid nitrogen prior to disruption using a mortar and pestle, with the exception of embryos, which were suspended in Trizol prior to disruption. All lysates were homogenized using Qiagen QIAshredder spin columns. Total RNA was isolated using the Qiagen RNeasy Mini Kit, following the protocol for animal tissues (Qiagen, Germantown, MD, USA). RNA was eluted in RNase-free water, and the quality and quantity were assessed with an Agilent 2100 Bioanalyzer (Agilent Technologies, Santa Clara, CA, USA). Two pools of RNAs were prepared for sequencing – pool 1 consisted of 1 μg RNA from each of embryos, 1^st^ instar nymphs, 2^nd^ instar nymphs, 3^rd^ instar nymphs, 4^th^ instar nymphs, and 5^th^ instar nymphs; pool 2 consisted of 1 μg RNA from each of an active male and female, as well as a male and female in diapause, for a combined total of 10 μg RNA.

### Library construction and sequencing

A strand-specific Illumina RNASeq library was prepared with the TruSeq RNA Sample Prep kit (Illumina, San Diego, CA, USA) with modifications to the manufacturer’s protocol. Second strand cDNA was synthesized with a dNTP mix containing dUTP, and after adapter ligation the second strand cDNA was digested to allow for strand-specific sequencing. The DNA was purified between enzymatic reactions and size selection of the library was performed with AMPure XT beads (Beckman Coulter Genomics, Danvers, MA, USA). The PCR amplification step was performed with primers containing a 7 nt index sequence. The library was enriched for low-abundance transcripts by treatment with double-stranded nuclease (DSN) (Evrogen, Moscow, Russia) following the procedure detailed in the DSN normalization application note (Illumina, San Diego, CA, USA). Libraries were sequenced using the 100 bp paired-end protocol on an Illumina HiSeq2000 sequencer. Raw sequencing data was processed using Illumina’s RTA and CASAVA for image analysis, base calling, sequence quality scoring, and index de-multiplexing. Data were then processed through FastQC and in-house pipelines for sequence assessment and quality control.

### De novo assembly of reads

Reads from all life stages were pooled and assembled using the Trinity package [17] version r2012-10-05 with the jellyfish k-mer method; RF strand-specific library type; jaccard clip; 200 bp minimum contig length; and paired_fragment_length of 400 bp.

### Identifying polymorphisms in the dataset

All sequencing reads were mapped against the putative transcripts using Bowtie2 and default parameters [54]. Duplicate reads were removed with Picard [55]. SAMtools MPILEUP [56] was then used allowing for 10 million reads per position. This file was then parsed to measure the base composition in positions with evidence for single nucleotide substitutions.

### Assessment of strand specificity of the library

We used a custom Perl script to parse the Bowtie2 mappings to identify properly mapped pairs by looking for the corresponding bit in the bitwise SAM flag. We then calculated the ratio of the number of these proper pairs in which the first-in-pair (read1) over the second-in-pair read (read2) mapped in the plus strand. This was log_2_-transformed and is denoted as the SSLR, which measures the strand specificity of the library. More specifically, the farther it is from zero, the more strand-specific the reads are underlying the putative transcript. Because of the strand-specific sequencing protocol of the library, read2 of each pair should be mapping on the sense (plus) strand of the mRNA making the SSLR negative for sense contigs. In contrast, positive values would indicate antisense contigs, some of which could be due to “leakage” in the sequencing protocol.

In the case of >80% overlap between two putative transcripts, as assessed by an all-vs-all BLASTN with cutoff of 10^−5^, the hits, range covered, and SSLR values for the two putative transcripts were examined. If the SSLR for the one putative transcript is < −2.5 and the other is >1.5, then these putative transcripts are considered as reverse complement of each other. In addition, if the length of the one having the positive SSLR was about the same, or shorter (<10% difference in length), compared to the putative transcript with the negative SSLR, then the one with the positive SSLR was excluded from post-processing and annotation, as it likely represents a redundant “leakage” transcript.

### Post-processing of the assembled putative transcripts

Some analyses require condensing multiple sequence variants output by Trinity down to a single, predominant transcript. In order to accomplish this, a filtering approach was applied that assigned an aggregate score to each putative transcript of a component (Table 1) based on numerous metrics. Each metric was normalized from 0–1 and had a different weight (Table 1). The putative transcript having the best score in each component was selected as representative for that component. The exact weights were determined iteratively. Additionally, after examining a histogram of all of the scores (Additional file 11: Figure S10), we required putative transcripts to have an aggregate score of >14, meaning they had a BLAST match or a predicted ORF. Only the putative transcript with the highest score was kept for each component, which was used in downstream analyses including functional annotation with the IGS annotation pipeline [57].

### BLASTX searches for post-processing

All of the Trinity putative transcripts were searched with BLASTX against reference proteins from the Uniref100 [58] and the non-redundant (NR) databases with an e-value of <10^−10^. A custom Perl script was used to calculate the percent match length of the protein with the best hit. Since Uniref is a high quality, curated database, the weights given for hits in Uniref are higher than those for NR. Since it is more important for a hit to cover a large fraction of the database protein than to cover a large fraction of the putative transcript, a higher weight is given for the match length relative to the reference protein when compared to the match length relative to the putative transcript. A hit that covers most of a protein in the database is more likely to represent a full-length transcript. Inversely, a hit covering most of the putative transcript, but poorly covering the reference protein is more likely to represent a partial transcript. The exact weights as well as the feature scored are shown in section A of Table 1. Of course, if a partial match is the only one available, it will still score best relative to all putative transcripts in the component.

### ORF prediction for post-processing

ORFs of at least 300 bp were identified on both strands in all three frames using (a) TransDecoder [17] and (b) Getorf from EMBOSS [59]. TransDecoder more accurately predicts start codons, compared to Getorf, because it is trained on the 500 longest ORFs, which are more likely to be real genes. As a result, predictions made by TransDecoder have a priority over the ORFs found by getorf. However, supplementing with Getorf predictions was important when identifying ORFs of bacterial origin, either from the metatranscriptome or from lateral gene transfer as they have properties that differ from the TransDecoder training set. We kept only the ORFs found in the strand in which the longest ORF is located. Additionally, if two ORFs overlapped for >50% of the length of the shorter putative transcript, only the longest of the two was retained.

Since the library is strand-specific, the ORFs predicted by TransDecoder in the plus strand of the putative transcripts were taken into account first. Subsequently, the TransDecoder minus strand ORFs that did not overlap with any TransDecoder plus strand ORFs were also added. Finally, ORFs found by the Getorf program in any strand were also added if they did not overlap with any TransDecoder ORF. A penalty is given if multiple ORFs are found in a transcript. An additional penalty occurs if the different ORFs have different best hits in Uniref100 and NR databases, or if they occur in both the plus and minus strand. All of these are indicative of incompletely spliced mRNAs or misassemblies.

### Coverage assessment for post-processing

To measure the evenness of the sequencing read coverage, all reads were mapped against the putative transcripts using Bowtie2. Given the presence of multiple sequence variants, the Bowtie2 parameter “-k 5000” was used in order to ensure that all mappings of each read are reported. In this way, all reads used for assembling each putative transcript were reported. SAMtools MPILEUP was used to obtain coverage for each position of a putative transcript and the mean coverage for each node calculated using a custom Perl script. A >5-fold difference in sequencing coverage of adjacent nodes in a putative transcript was considered a coverage dip. Sequencing coverage dips can be signs of misassemblies or alternative splicing. Therefore, it is important to consider that this step may remove valid transcripts in order to generate a representative set of transcripts for annotation.

### Annotation

ORFs that were predicted by TransDecoder [17] and Getorf from EMBOSS [59] were assigned putative function with the IGS Eukaryotic Functional Annotation Pipeline protocol 2 in a manner analogous to the IGS Prokaryotic Functional Annotation Pipeline [57]. ORFs were searched against an HMM library comprising models from TIGRFAMs 13.0 [60, 61] and PFAM 26.0 [62] using HMMER3 [63]. ORFs received the annotation associated with that HMM for all equivalog-level matches. Subsequently, ORFs without a significant HMM match were annotated using a homologous sequence (e-value <1e-30) identified with BLAST against UniProtKB/SwissProt [64], a curated protein database. ORFs with no HMM or BLAST matches were annotated as “hypothetical protein". ORFs with annotation were associated with appropriate gene symbols, E.C. numbers, and GO terms from a large collection of annotation assertions that are associated with the HMMs and proteins identified through BLAST.

### Differential gene expression

The sequencing reads from each pool were aligned separately to the Trinity assembly with TopHat v1.4.0 [65] allowing for up to 2 mismatches per 25 bp segment and removing reads that aligned to more than 20 genomic locations. Duplicates were removed with Picard [55], coverage calculated with SAMtools MPILEUP [56], and read counts per transcript calculated with a local Perl script. For the 61,606 genes with >50 reads per transcript, the log_2_((Coverage Tube A1)/(Coverage Tube B2)) was calculated for each transcript and normalized by centering the mode of a histogram with intervals of 0.1, as described previously [66]. Putative transcripts with no reads mapped were given the value of 0.5 to enable analysis of transcripts present in only one pool.

### qRT-PCR

Primers were designed based on putative transcripts in order to determine transcript abundance for genes with similarity to a known bLys, *Wolbachia* ankyrin genes, and to constitutively expressed aphid genes (Additional file 12: Table S2). Primers were designed with Primer3 and synthesized by Sigma-Aldrich (St. Louis, MO, USA). The same RNA from each of ten life stages that was used for pooling was now used separately as a template in three replicate transcription reactions, performed with the QuantiTect Reverse Transcription Kit (Qiagen, Germantown, MD, USA) following the manufacturer’s instructions. Specifically, 1 μg of total RNA was incubated with gDNA Wipeout Buffer (7×) and RNase-free water at 42°C for 2 min to remove contaminating genomic DNA. The cDNA was synthesized from the RNA using Quantiscript Reverse Transcriptase (RT), Quantiscript RT Buffer (5×), and RT Primer Mix at 42°C for 30 min and then at 95°C for 3 min to inactivate the Quantiscript RT. Dilutions of the cDNA (0.5 μL of cDNA per 25 μL reaction) were used as templates in a qPCR reaction containing QuantiTect 2× SYBR Green PCR Master Mix, gene-specific primers, and RNase-free water, using the standard protocol. The assays were conducted using an ABI 7900HT instrument (Applied Biosystems, Foster City, CA, USA). The reactions were denatured at 95°C for 15 min followed by amplification with 45 cycles of 94°C for 15 s, 55°C for 30 s, and 72°C for 30 s. Reactions were followed by a melt curve analysis starting at 55°C, with a dissociation step at 95°C for 1 min plus 0.5°C/cycle for 80 cycles. Data was analyzed by comparing the Ct values for the eight loci of bacterial ancestry with the average Ct of four BMSB loci thought to be constitutively expressed.

### Testing for presence of *Wolbachia*endosymbionts

A previously described set of 42 degenerate primers [33, 67] were used to test for the presence of *Wolbachia* DNA in the stink bug genome. Previously, these primers were used successfully to amplify *Wolbachia* loci in divergent host species [33]. Genomic DNA was isolated from adult stink bugs using the Qiagen DNeasy Blood & Tissue Kit following the protocol for animal tissues (Qiagen, Valencia, CA, USA) and quantified using a Quanti-iT PicoGreen dsDNA Kit (Life Technologies, Carlsbad, CA, USA). Following DNA isolation, the 42 degenerate primers were used in a PCR reaction using Qiagen HotStarTaq DNA Polymerase with activation at 95°C for 15 min, followed by amplification with 45 cycles of 94°C for 30 s, 55°C for 30 s, and 72°C for 1 min, with a final extension of 72 °C for 10 min. As a positive control for PCR, a subset of 12 degenerate primers were used in PCR on tetracycline-treated *Drosophila ananassae* Hawaii isolate DNA, whose genome has previously been shown to contain *Wolbachia* DNA [67]. Aliquots of 10 μL of the amplicons were run on a 1.5% agarose gel and examined with a UV transilluminator (Bio-Rad, Hercules, CA, USA).

### Phylogenies

Homologous proteins for phylogenetic analysis were identified using a BLASTX [35] search of NR and the NCBI whole transcriptome database. Protein sequences were aligned using CLUSTALW [36], except for lysozyme which was aligned with MAFFT [40]. Maximum likelihood phylogenies were generated using the rapid Bootstrap analysis and search for a best-scoring ML tree in one run as implemented in RAxML [37] using the PROTGAMMA model with a BLOSUM62 matrix with 100 alternative runs on distant starting trees and ‘12345’ as the specified integer for parsimony and bootstrapping analyses, with the exception of lysozyme, mannanase, and amylase genes where 1000 alternative runs were used. Bootstrap values <60% were removed from the figures.

## Availability of supporting data

The sequencing reads are available from the Sequence Read Archive (SRA) under the accessions SRX554889 and SRX554890. The transcriptome shotgun assembly has been deposited at DDBJ/EMBL/GenBank under the accession GBHT00000000. Intermediate analysis files and Perl scripts are available upon request for others seeking to apply these criteria to their transcriptome analysis.

## Electronic supplementary material

Additional file 1: Figure S1: Frequency of ORFs relative to the SSLR. The number of ORFs found in the plus and the minus strand of putative transcripts that were predicted to be either sense or anti-sense, based on the log_2_(read1/read2) metric. (ZIP 114 KB)

Additional file 2: Figure S2: The log2(read1/read2 in plus strand) distribution for the putative transcripts. A titration was done using different cutoffs for the number reads mapping to each putative transcript. (ZIP 198 KB)

Additional file 3: Table S1: Differential Expression between Adults and Pre-adults. An Excel spreadsheet that lists (A) all of the putative transcripts by name, (B) their annotation, (C) the aggregate read coverage for a putative transcript for pre-adult life stages, (D) aggregate read coverage for a putative transcript for adult life stages, and (E) the normalized log_2_Ratio. A value of "0.5" in columns C & D means no reads were found but allowed for a normalized log ratio to be calculated. (XLSX 3 MB)

Additional file 4: Figure S3: Krona plot of all unfiltered putative transcripts. The lowest common ancestor for the best BLASTX match(es) to NR for each transcript is visualized in a Krona plot for the unfiltered putative transcripts. (ZIP 400 KB)

Additional file 5: Figure S4: Krona plot for only bacterial reads in the unfiltered putative transcripts. The lowest common ancestor (LCA) for the best BLASTX match(es) to NR for each transcript is visualized in a Krona plot for the unfiltered reads with a bacterial LCA. (ZIP 291 KB)

Additional file 6: Figure S5: Krona plot of all filtered putative transcripts. The lowest common ancestor for the best BLASTX match(es) to NR for each transcript is visualized in a Krona plot for the filtered reads. (ZIP 351 KB)

Additional file 7: Figure S6: Krona plot for only bacterial reads in the filtered putative transcripts. The lowest common ancestor for the best BLASTX match(es) to NR for each transcript is visualized in a Krona plot for the filtered reads having a bacterial lowest common ancestor. (ZIP 328 KB)

Additional file 8: Figure S7: Phylogeny of comp2753_c5_seq1. This transcript had significant matches to ankyrin proteins encoded by *Wolbachia* endosymbionts (α-Proteobacteria) and *Diplorickettsia massiliensis* (α-Proteobacteria). The numbers are the GenBank GI numbers. (ZIP 103 KB)

Additional file 9: Figure S8: Phylogeny of comp549_c15_seq3. This transcript had significant matches to ankyrin proteins encoded in 95 *Wolbachia* endosymbionts (α-Proteobacteria), *Rickettsiella grylii* (γ-Proteobacteria), and *Diplorickettsia massiliensis* (α-Proteobacteria), all obligate intracellular bacteria that infect arthropods. The presence of this CDS in multiple diverse taxa suggests several independent lateral gene transfers. The numbers are the GenBank GI numbers. (ZIP 106 KB)

Additional file 10: Figure S9: Mannanase N-terminus Phylogeny. Phylogenetic analysis of the N-terminus of the putative mannanase proteins reveals a well-supported clade whose relationship is not well resolved with respect to the bacterial homologues examined. (ZIP 216 KB)

Additional file 11: Figure S10: Distribution of scores assigned to each, non-reverse complementary putative transcript. Putative transcripts with a score <14 were filtered out of the dataset, which includes those putative transcripts that did not have a BLAST match or a predicted ORFs. (ZIP 108 KB)

Additional file 12: Table S2: qRT-PCR and qPCR primer pairs. (DOC 48 KB)

## Abbreviations

BMSB: Brown Marmorated Stink Bug
COG: Cluster of orthologous groups
LCA: Lowest common ancestor
SSLR: Strand-specific log ratio.
