# Supplementary material for: Rapid transcriptome sequencing of an invasive pest, the brown marmorated stink bug Halyomorpha halys
Source: BMC Genomics. 2014 Aug 29;15(1):738. doi: 10.1186/1471-2164-15-738 (PMC4174608; doi:10.1186/1471-2164-15-738)
Supplement: Supplementary file 12 — Additional file 12: Table S2: qRT-PCR and qPCR primer pairs. (DOC 48 KB) [file 12864_2014_6471_MOESM12_ESM.doc]

## Table S2. qRT-PCR and qPCR primer pairs

| **Putative transcript** | **Functional annotation** | **F-primer** | **R-primer** | **Size (bp)** |
| --- | --- | --- | --- | --- |
| comp17945_c0_seq3 | L27 ribosomal protein | CTTCGGGTGAACCAAATTCTC | AGCTTGACAGTAAGGACATCACG | 148 |
| comp6396_c1_seq1 | L30 ribosomal protein | CTAACGGAGAGTTTGTTGTTTCG | GGCTCATTCCATTTATTTCTGG | 139 |
| comp4381_c3_seq1 | bLys | AATTCAAGCAGAGAAGGAAGGTC | TCTTCCACATCCAACATCAGTAG | 138 |
| comp4662_c1_seq1 | bLys | TTACTATGAGAGGCGGATAGAGG | CAGATCCAGGACTAGAAGAATGC | 148 |
| comp2913_c1_seq1 | Tubulin beta-1 | GATACCTTTGGAGAGGGAATGAC | GTTTCAACTCACCCACTCGTTAG | 132 |
| comp4674_c0_seq1 | Alanyl-tRNA synthetase | ATTCTGCATATTGGCGTTGTTG | GTGTGTTGCTGTATGGTTGCTC | 111 |
| comp3672_c1_seq2 | Threonyl-tRNA synthetase | GCAACACCCTTTGATGATTATG | TACTGAGCTAATTGGGCATTTC | 131 |
| comp2753_c5_seq1 | Homology to ankyrin gene | CTTTCATCAGTCGGTTCCTCTC | ATCCCGATGTCTACTGCATCTAC | 145 |
| comp18511_c0_seq1 | Homology to ankyrin gene | GTCTCTTATCGGGACGAATGTG | GCCGAAACTTCTTCTTCTATAGGC | 149 |
| comp549_c15_seq3 | Homology to ankyrin gene | CTCCAAGACACCAGCTTATCTTC | CGTCTTCGCTTTCTCAATGTTATC | 148 |
| comp17945_c0_seq4 | Constitutively expressed | CTTCGGGTGAACCAAATTCTC | AGCTTGACAGTAAGGACATCACG | 148 |
| comp6396_c1_seq1 | Constitutively expressed | CTAACGGAGAGTTTGTTGTTTCG | GGCTCATTCCATTTATTTCTGG | 139 |
| comp4381_c3_seq2 | bLys | AATTCAAGCAGAGAAGGAAGGTC | TCTTCCACATCCAACATCAGTAG | 138 |
| comp4662_c1_seq1 | bLys | TTACTATGAGAGGCGGATAGAGG | CAGATCCAGGACTAGAAGAATGC | 148 |
| comp29721_c0_seq2 | Tubulin beta-1 | GATACCTTTGGAGAGGGAATGAC | GTTTCAACTCACCCACTCGTTAG | 132 |
| comp4674_c0_seq1 | Alanyl-tRNA synthetase | ATTCTGCATATTGGCGTTGTTG | GTGTGTTGCTGTATGGTTGCTC | 111 |
| comp3672_c1_seq1 | Threonyl-tRNA synthetase | GCAACACCCTTTGATGATTATG | TACTGAGCTAATTGGGCATTTC | 131 |
| comp18611_c0_seq1 | Similarity to a Wolbachia ankyrin gene (CAQ54400.1) | ACGTTCTTCTGGATCTCTGATTG | AAGAGGCAAGCACCGAAATG | 150 |
| comp2753_c5_seq1 | Similar to a Wolbachia ankyrin gene (EEB55313.1) | CTTTCATCAGTCGGTTCCTCTC | ATCCCGATGTCTACTGCATCTAC | 145 |
| comp6503_c0_seq1 | Similar to a Wolbachia ankyrin gene (ZP_03335447.1) | CAAATGTTATGCCTTCTTCGAG | GAAGGAGGAGAATCAGTAATTGG | 127 |
| comp18511_c0_seq1 | Similar to a Wolbachia ankyrin gene (ZP_03335447.1) | GTCTCTTATCGGGACGAATGTG | GCCGAAACTTCTTCTTCTATAGGC | 149 |
| comp549_c15_seq3 | Similar to a Wolbachia ankyrin gene (CAQ54400.1) | CTCCAAGACACCAGCTTATCTTC | CGTCTTCGCTTTCTCAATGTTATC | 148 |
| comp7015_c0_seq1 | Mannanase | TGCTCAGATGTTCTTTAATGTCAG | ACGAAACCATTGCCTAATCC | 128 |
| comp11444_c0_seq1 | Mannanase | TGTTCACCTTCCCAAAGGAC | TTGTTCTTGCATTTGCGTTG | 126 |
| comp2467_c6_seq1 | Mannanase | TGAGGAACATGGAGTTGAAGG | TCAACAGCTTCCTCGTGTTG | 114 |
| comp1411_c3_seq2 | Amylase | ATCTCCAGTCATTGGGCATC | TCCAATGTTCCGTATTCTGG | 121 |
| comp15321_c0_seq1 | Endosymbiont gene | CCACGGTCCTGGTAGCTTTAC | GCTGCTATTTGGTTCCAAGC | 138 |
| comp26460_c0_seq1 | Endosymbiont gene | GGGAAGCGAGAGCTTACGAC | CCAGGTGGAGTTTACCATGC | 147 |
| comp27004_c0_seq1 | Endosymbiont gene | TGCCTGTTGCATTATTGCTC | CAATCTTAGTTGAACCAGTGTCG | 141 |
| comp16196_c0_seq1 | Endosymbiont gene | ATGGAACGGTCTGGAAAGTC | CCCATATTCAGACAGGATACCAC | 114 |
